# Systematic reviews of health economic evaluations: a protocol for a systematic review of characteristics and methods applied

**DOI:** 10.1186/s13643-017-0639-8

**Published:** 2017-12-02

**Authors:** Miriam Luhnen, Barbara Prediger, Edmund A. M. Neugebauer, Tim Mathes

**Affiliations:** 1 Department Health Care and Health Economics, Institute for Quality and Efficiency in Health Care (IQWiG), Im Mediapark 8, 50670 Cologne, Germany; 2 Faculty of Health, Department of Medicine, Witten/Herdecke University, Ostmerheimer Str. 200, Haus 38, 51109 Cologne, Germany; 3 Institute for Research in Operative Medicine, Witten/Herdecke University, Ostmerheimer Str. 200, Haus 38, 51109 Cologne, Germany; 4 Faculty of Health, Brandenburg Medical School – Theodor Fontane, Campus Neuruppin, Fehrbelliner Str. 38, 16816 Neuruppin, Germany; 5 Interdisciplinary Centre for Health Services Research, Witten/Herdecke University, Alfred-Herrhausen-Straße 50, 58448 Witten, Germany

**Keywords:** Systematic review, Economic evaluation, Reimbursement, Medical decision making

## Abstract

**Background:**

The number of systematic reviews of economic evaluations is steadily increasing. This is probably related to the continuing pressure on health budgets worldwide which makes an efficient resource allocation increasingly crucial. In particular in recent years, the introduction of several high-cost interventions presents enormous challenges regarding universal accessibility and sustainability of health care systems. An increasing number of health authorities, inter alia, feel the need for analyzing economic evidence.

Economic evidence might effectively be generated by means of systematic reviews. Nevertheless, no standard methods seem to exist for their preparation so far.

The objective of this study was to analyze the methods applied for systematic reviews of health economic evaluations (SR-HE) with a focus on the identification of common challenges.

**Methods/design:**

The planned study is a systematic review of the characteristics and methods actually applied in SR-HE. We will combine validated search filters developed for the retrieval of economic evaluations and systematic reviews to identify relevant studies in MEDLINE (via Ovid, 2015-present). To be eligible for inclusion, studies have to conduct a systematic review of full economic evaluations. Articles focusing exclusively on methodological aspects and secondary publications of health technology assessment (HTA) reports will be excluded. Two reviewers will independently assess titles and abstracts and then full-texts of studies for eligibility. Methodological features will be extracted in a standardized, beforehand piloted data extraction form. Data will be summarized with descriptive statistical measures and systematically analyzed focusing on differences/similarities and methodological weaknesses.

**Discussion:**

The systematic review will provide a detailed overview of characteristics of SR-HE and the applied methods. Differences and methodological shortcomings will be detected and their implications will be discussed. The findings of our study can improve the recommendations on the preparation of SR-HE. This can increase the acceptance and usefulness of systematic reviews in health economics for researchers and medical decision makers.

**Systematic review registration:**

The review will not be registered with PROSPERO as it does not meet the eligibility criterion of dealing with clinical outcomes.

**Electronic supplementary material:**

The online version of this article (10.1186/s13643-017-0639-8) contains supplementary material, which is available to authorized users.

## Background

Continuing pressure on health budgets worldwide makes an efficient resource allocation increasingly crucial. In recent years, the introduction of several high-cost interventions presents enormous challenges regarding accessibility and sustainability of health care systems [1, 2]. This makes economic considerations more important for health authorities and their decision-making process regarding pricing and reimbursement especially of new interventions.

Systematic reviews of health economic evaluations (SR-HE) can provide evidence about the cost-effectiveness of an intervention within a limited time frame. They are valuable (1) to inform the development of an own economic model, (2) to identify the most relevant studies for a particular decision, and (3) to identify the implicated economic trade-offs [3]. Moreover, provided that high-quality economic evaluations that exist are sufficiently transferable and demonstrate similar results regarding cost-effectiveness, SR-HE might indicate the most cost-effective intervention.

Jefferson et al. [4] found that SR-HE show fundamental methodological flaws, especially regarding their search strategy and the application of an appropriate quality assessment tool. Nevertheless, little research has been performed to further develop the methods for SR-HE in the meantime. Standards for the preparation of SR-HE do not seem to exist so far: More recent studies focusing on the available methodological guidelines found that the recommendations still vary widely and are partly imprecise [5–8]. It is therefore to be expected that the conduct of SR-HE still varies widely and still shows methodological shortcomings. The aim of this paper is

- To provide a detailed overview of the characteristics and applied methods in recently published SR-HE
- To identify similarities and differences between the characteristics and methods of SR-HE
- To identify common challenges

## Methods/Design

### Protocol

We used the PRISMA-P (Preferred Reporting Items for Systematic review and Meta-Analysis Protocols) 2015 checklist to develop the methods for this systematic review protocol [9] (please see Additional file 1).

Should protocol amendments be necessary, these will be documented including details of the date, changes made, and the rationale for changes.

### Literature search

A systematic search in Ovid MEDLINE(R) Epub Ahead of Print, In-Process & Other Non-Indexed Citations, Ovid MEDLINE(R) Daily, and Ovid MEDLINE(R) 1946 to Present will be performed. We will limit the publication date of our search to the period 2015/01/01 to present. A validated search filter for economic evaluations (Emory University [Grady] [10]) will be combined with a validated filter for the retrieval of systematic reviews (Lee [11]), as presented in Table 1. This strategy was chosen as it provides an optimal balance between sensitivity and precision. Search results will be downloaded to EndNote version X7 where duplicates will be identified and removed.

**Table 1:** Details of the bibliographic database search strategy

Step	Search string	Reference
1	((economic$.ti. or cost$.ti. or cost benefit analysis/ or (treatment outcome/ and ec.fs.)) not ((animals/ not humans/) or letter.pt.))	Emory University (Grady) [10]
2	MEDLINE.tw. or systematic review.tw. or meta-analysis.pt. or intervention$.ti.	Lee 2012 [11]
3	1 and 2	

Limit: publication year 2015–present

### Inclusion criteria

We will include articles available as full-text and written in English, German, French, or Spanish if they fulfill all of the following criteria:

1. Systematic literature search in at least one electronic database and transparent description of study selection. We will exclude articles applying abbreviated review methods (e.g., scoping reviews and short reviews) as judged by the authors of the SR-HE.
2. Inclusion of full economic evaluations (i.e., cost-effectiveness/cost-utility/cost-benefit-analyses [12]) and/or the cost-effectiveness of an intervention was reviewed. Articles reviewing solely partial economic evaluations (like cost-of-illness studies or budget impact analyses) will be excluded.
3. Objective to answer a cost-effectiveness research question, i.e., we will exclude articles focusing exclusively on methodological aspects (e.g., analysis of methods applied in health economic modeling studies).
4. Full-text journal article. Protocols, commentaries, editorials, and conference proceedings will be excluded. Likewise, secondary publications of HTA reports will be excluded as the focus of our study will be on the scientific literature instead of documents stemming from regulatory processes within a certain jurisdiction in a health care system.

### Study selection

Two reviewers will independently assess the titles and abstracts retrieved in the electronic literature search against the inclusion criteria. Possible eligible full-text articles will be retrieved and screened by two reviewers to reach a final decision about inclusion. Any disagreements will be resolved through discussion or involvement of a third reviewer.

We will prepare a PRISMA flowchart to illustrate the selection process.

### Data abstraction

Methodological features will be extracted in a standardized, beforehand piloted data extraction form (Table 2). We developed an electronical extraction form in Microsoft Excel 2010 for a previous study (not published yet) in which we analyzed HTA reports of international HTA organizations for the methods applied for SR-HE and adapted it for the purpose of the present study. This approach for data abstraction and data presentation was inspired by the publication of Page et al. [13] which provides an overview of epidemiology and reporting characteristics of systematic reviews of biomedical research. Data items presented in the included articles will be classified according to the categories depicted in Table 3. Data will be extracted each by a single reviewer. After extraction of the first articles, a 10% random sample will be verified for accuracy and correctness of data entries by a second reviewer. Discrepancies will be resolved through discussion or third party, if necessary. In case of frequent and/or substantial disagreements, a verification of 100% is intended.

**Table 2:** Data extraction form

Article
General information
Affiliation (e.g., academic, commercial, public)
Country of corresponding author
Number of authors
Journal + impact factor
Disease area(s) (ICD-Code[s])
Type of intervention (e.g., drug treatment, surgical procedure)
Scope of SR-HE (only SR-HE/SR-HE and primary CEA/SR-HE to inform primary CEA)
If only SR-HE: indicated purpose of systematic review
Study registered or published protocol available (not stated/stated)
Consideration of reporting guideline (e.g., PRISMA)
Statement of research question and formulated eligibility criteria
Research question (not stated/stated)
Eligibility criteria (PICOS + further [specify])
Economic study types included
Literature search strategy
Information sources (databases, reference lists of relevant records, etc.)
Search terms/filters + explanation when economic terms missing (e.g., joint review for clinical and economic effectiveness)
Search limits (time period, language, publication type, etc.)
Study selection
Flow of study selection described (yes/no)
Study selection illustrated in flow chart (yes/no)
Duplicate study selection (yes/no/unclear) + method (e.g., all independently/quality assurance of sample) + mechanism to resolve disagreement
Technical support for study selection (e.g., software)
Data extraction
Data extraction method (e.g., standardized data extraction form)
Duplicate data extraction (yes/no/unclear) + method (e.g., all independently/quality assurance of sample) + mechanism to resolve disagreement
Data items extracted
Technical support for data extraction (e.g., software)
Assessment of methodological study quality
Assessment of methodological study quality on study level (yes/no/unclear) + assessment tool
Duplicate quality assessment (yes/no/unclear) + method (e.g., all independently/quality assurance of sample) + mechanism to resolve disagreement
Assessment of generalizability/transferability/applicability
Assessment of generalizability/transferability/applicability (yes/no/unclear) + assessment tool
Duplicate generalizability/transferability/applicability assessment (yes/no/unclear) + method (e.g., all independently/quality assurance of sample) + mechanism to resolve disagreement
Presentation of cost data
Presentation of cost data (as reported/inflated/currency converted)
Method for data synthesis
Data synthesis
Further remarks

*CEA* cost-effectiveness analysis; *PICOS* patient, intervention, comparison, outcome, setting; *PRISMA* Preferred Reporting Items for Systematic review and Meta-Analysis; SR-HE systematic review of health economic evaluations

**Table 3:** Categories for the classification of data items extracted in the included reviews

Category	Data item	Presented/reported
Study details		
	Author(s)	
	Year of publication	
	Objective	
	Country	
	Setting	
	Funding	
Methods		
	Population	
	Intervention(s)	
	Comparator(s)	
	Outcomes/effects	
	Study design	
	PICO	
	PICOS	
	Methods for valuing outcomes/benefits	
	Model type	
	Perspective	
	Time horizon	
	Cost/resource items included	
	Data sources for costs	
	Data sources for clinical data	
	Data sources for utility data	
	Discounting	
	Currency	
	Analysis of uncertainty	
Results		
	Costs/resources	
	Outcomes/benefits	
	Incremental	
	Analysis of uncertainty	
	Author’s conclusion	

### Data analysis and presentation

We will analyze all data using Microsoft Excel 2010. Results for each data item extracted will be presented in tables. For nominal data, we will provide numbers and percentages. We will provide median and ranges for ordinal data.

In order to allow an estimation of the number of SR-HE published per year and to analyze possible changes over time, we will present the number of hits resulting from our search strategy for the years 2015 to 2017.

Since no tool for the critical appraisal of SR-HE exists (comparable e.g., to AMSTAR [A Measurement Tool to Assess Systematic Reviews] [14]), we will not critically appraise included articles by means of a certain tool but focus on similarities, differences, and methodological shortcomings.

As far as possible, the results of our study will be reported in accordance with the PRISMA guidelines [15].

## Discussion

The systematic review will provide a detailed overview of characteristics of SR-HE and the applied methods. Differences and methodological shortcomings will be detected and their implications will be discussed. The findings of our study can improve the recommendations on the preparation of SR-HE. This can increase the acceptance and usefulness of systematic reviews in health economics for researchers and medical decision makers.

## Additional file

Additional file 1: Preferred Reporting Items for Systematic review and Meta-Analysis Protocols (PRISMA-P) 2015 checklist: recommended items to address in a systematic review protocol. (DOCX 36 kb)

## Abbreviations

AMSTAR: A Measurement Tool to Assess Systematic Reviews
CEA: Cost-effectiveness analysis
PICOS: Patient, intervention, comparison, outcome, setting
PRISMA: Preferred Reporting Items for Systematic review and Meta-Analysis
PROSPERO: Prospective Register of Systematic reviews
SR-HE: Systematic reviews of health economic evaluations
